# Supplementary material for: Are carbon emissions trading and green financial instruments synergistic? -Comprehensive quantitative research based on content analysis
Source: PLoS One. 2024 Mar 7;19(3):e0298601. doi: 10.1371/journal.pone.0298601 (PMC10919601; doi:10.1371/journal.pone.0298601)
Supplement: S1 Data — (ZIP) [file pone.0298601.s001.zip › Data/地级市Z_F工作报告.zip!/╘╞─╧/└Ñ├≈╩╨/└┤╫╘╣1⁄2╓┌║┼í╛┬φ┐╦╩2╛▌═°í┐_2017.docx]

各位代表：

我代表市人民政府，向大会报告工作，请予审议，并请各位政协委员提出意见。

一、 2016年及市第十三届人民政府工作回顾

过去的一年，在省委、省政府和市委的坚强领导下，在市人大及其常委会和市政协的监督支持下，我们坚持稳中求进，积极推进供给侧结构性改革，攻坚克难、真抓实干，圆满完成了市十三届人大七次会议确定的目标任务，实现了“十三五”良好开局。全市实现地区生产总值4300亿元，增长8.5%，其中，一、二、三产业增加值分别增长6%、7.6%、9.3%；一般公共预算收入530亿元，增长5.5%；城镇和农村常住居民人均可支配收入分别达36739元、12555元，分别增长8.2%、9.7%。主要抓了七个方面的工作：

（一）全力以赴稳增长，经济发展稳中有进。坚持把稳增长作为首要任务，认真贯彻落实国家、省稳增长政策，及时出台市稳增长46条、提振实体经济19条等举措，经济运行逐季向好、企稳回升。加大力度增投资。狠抓项目前期工作，市县共安排项目前期费4.6亿元，固定资产投资三年滚动计划项目库不断充实。落实投资包保责任制、定期会办制、现场办公制，协调解决建设难题，黄马高速等121个在建项目加快推进。构建多元化投融资格局，推动设立500亿元昆明合作发展基金、750亿元“五网”建设基金，争取国家专项建设基金116.7亿元，新增政府债券44.6亿元，启动福宜高速公路等14个总投资652亿元的PPP示范项目。全市规模以上固定资产投资3920亿元，增长12.1%。挖掘潜力扩消费。加快发展农村电商、跨境电商，消费新热点新业态不断涌现，阿里巴巴·昆明产业带平台、宜良农村淘宝、石林乐村淘上线运营，禄劝成为国家级电子商务进农村综合示范县，社会消费品零售总额2310亿元，增长12.1%。全域旅游提速发展，安宁奥特莱斯风情小镇一期等建成开业，古滇文化旅游名城获评“国家级生态旅游示范区”，旅游总收入突破千亿元，达1073.5亿元、增长48.4%。成为国家“十三五”服务业综合改革试点城市、国家文化消费试点城市。千方百计扶工业。通过“股权+债权”“财园助企贷”等方式，破解企业融资难融资贵问题，落实企业帮扶资金超过14亿元，成立13个市级工作组结对帮扶重点企业，昆宝电缆等103户重点企业恢复生产或扩大生产。空港科技创新园等51个亿元以上项目开工，昆客搬迁技改等45个项目竣工，中石油云南炼油项目点火试车，新建投产规模以上工业企业52户，规模以上工业增加值增长4.5%。提质增效促农业。蔬菜、花卉、中药材、烤烟等特色产业效益持续增长，新建西山自耕农庄等都市农庄9座，新增省级农业龙头企业15户，10个重点农业园区产值达30亿元，增长9.1%。提振信心稳地产。实施房地产健康发展24条措施，建立促进房地产业发展联动机制，设立200亿元保障房以购代建基金，保障性安居工程货币化安置3000套，房地产投资完成1530.5亿元，增长5.5%，商品房销售面积达1521万平方米，增长16.5%。

（二）实施创新驱动，新动能加快形成。结构调整步伐加快。启动“188”重点产业建设，编制了19个重点产业规划，出台了实施意见，建立了“五个一”工作机制，全市产业发展和结构调整的方向重点更加明确。电子信息、生物医药、先进装备制造业增加值分别增长32.2%、21.7%、8%，均高于一般工业增速。新兴产业培育壮大。呈贡信息产业园完成投资17亿元，云南省信息化中心、浪潮昆明云计算产业园等项目加快建设，中兴通讯等40个项目签约入驻。举办昆明大健康国际高峰论坛，发布大健康产业发展规划，宝相健康产业园启动建设，成为国家医养结合试点城市。金鼎科技园成为国家级广告产业园，凤凰纵横文旅等11个项目落地，C86山茶坊文化艺术园等项目建成。楼宇总部经济加快发展，中铁建西南总部、中国铜业等企业总部落户昆明。创业创新蓬勃发展。组建双创联盟，建立创客合作银行，与昆明理工大学等5所高校共建双创学院，云上小镇建设初见成效，建成国家级、省级大学生创业示范园23个，省级众创空间、市级新型双创孵化园26个，猪八戒网、清华启迪等一批知名双创企业落户昆明，成功申报为全国小微企业创业创新基地示范城市，呈贡区、经开区等列入全省首批双创示范基地。新认定高新技术企业126家，高技术制造业增加值增长17.8%。专利申请和授权量突破2万件，全社会研发投入占GDP比重达2.1%。

（三）全面发力“五网”建设，承载能力不断增强。启动“五网”建设五年大会战，实施项目205项，完成投资791.4亿元。成为全国首批综合交通枢纽示范城市。路网加快建设。石林至泸西等6条高速公路开工，呈澄、小龙高速建成通车，绕城高速东南段、功东高速等顺利推进，在建高速公路项目16个、659公里。地铁1号线呈贡支线投入运营，1号线西北延线、2号线二期、3号线、4号线、5号线、6号线二期、9号线一期加快建设，在建里程137公里。7号线、8号线、嵩明线、安宁线试验段动工。联大立交通车，完成王家营等4座立交改造提升，主城交通连接更加顺畅便捷。航空网加密延伸。长水机场综合交通枢纽建设稳步推进，东川、安宁等4个通用机场启动建设，新增国内外航线24条。能源网加快推进。建成天然气支线管道10公里、城市燃气管道145公里，完成天然气置换48.8万户。建成新能源汽车充电桩1014个。富民金铜盆、寻甸苏撒坡等风电场项目竣工。水网不断完善。鱼龙、箐门口等9件中小型水库开工建设，完成532件农村饮水安全巩固提升工程，16万人受益，3万件“五小水利”工程投入使用，新增和改善灌溉面积8.8万亩。互联网覆盖提升。昆明国际通信枢纽加快建设，城区20M宽带互联网覆盖率超过80%，行政村宽带光纤覆盖率超过90%，4G网络基本覆盖。

（四）加强人居环境建设，城市品质不断提升。规划体系进一步完善。《昆明市城市总体规划（2011—2020年）》获国务院批复，编制《昆明“十三五”城乡发展规划》《翠湖周边历史文化片区整治提升规划》等专项规划，滇池流域地区基本完成“多规合一”工作。开发建设有序推进。草海、巫家坝等重点片区规划建设有序推进。呈贡新区功能不断完善。重新启动16个城中村改造项目。新开工城市道路91条，续建126条，完成道路综合整治19条，主城区新增停车泊位2.2万个。城乡人居环境提升工作全面开展。统筹推进“四治三改一拆一增”“七改三清”环境整治，推行环卫一体化试点，拆除临违建筑433万平方米，新建改造城市和农村公厕1606座，1188座城市公厕免费开放。新增城市绿地544.9公顷，新增造林26.7万亩。建成77个乡镇农村污水收集处理设施，完成通村公路路面硬化1186公里、路基改造438公里，建成85个省级美丽宜居乡村示范村。主城区空气质量优良率达98.9%，万元生产总值能耗下降9%以上。水环境整治取得新成效。滇池保护治理完成投资24.3亿元，新建污水管网109公里，退塘还湖960亩，底泥疏浚75万立方米，纳入国考的12条入滇河道水质全部达标，滇池治理取得国家年度考核良好成绩，水质由劣Ⅴ类提升为Ⅴ类，为20年来最好水平。阳宗海水质总体达Ⅲ类，牛栏江出境断面平均水质达Ⅱ类。

（五）深入推进改革开放，发展活力得到释放。供给侧结构性改革扎实推进。化解煤炭产能28万吨、钢铁产能150万吨。商品房库存下降23.9%，住宅去库存周期进一步缩短。与17家金融机构建立银政战略合作关系。置换债券610亿元，综合融资成本3%，实施过渡性融资300多亿元，综合融资成本控制在6%以下，债务期限结构不断优化，融资成本明显降低。成立昆明售电有限公司，市场化交易电量153亿千瓦时，实实在在为昆明的企业降低用电成本约19亿元。关键环节改革取得实效。深化“放管服”改革，出台38条创造一流投资服务环境措施，实施政务服务第三方评价，行政审批网上服务大厅、投资审批中介超市、市场主体信用信息服务监管平台上线运行，成为国家公共资源交易电子化招投标试点城市。“双随机、一公开”全面推行。寻甸县、嵩明县信用联社在全省首批改制成为农商银行。完成党政机关公务用车改革。“五证合一、一照一码”登记制度全面实施。启动不动产统一登记。农村土地承包经营权确权登记颁证工作持续深入，国有林场改革取得实效。文化、社会、生态等领域改革有序推进。开放水平不断提升。成功举办“昆明市·滇中新区开放合作推介会”等系列活动，大健康3D打印产业园等一批优质项目签约落地，引进市外到位资金915.3亿元，增长10.5%，实际利用外资7.4亿美元，增长54.7%。开放平台载体进一步夯实，昆明综合保税区一年建成，即将迎接国家封关验收。昆蓉欧班列双向开行，中亚铁海联运国际货运班列开通，跨境多式联运通道建设取得突破。成功举办第4届南博会、第14届中国农交会、第74届中国药交会、中印瑜伽大会等重大活动。法国、德国等15国在昆设立签证中心。亚洲财富论坛永久落户昆明。

（六）补齐民生短板，人民生活不断改善。加大财政民生投入，民生支出达500亿元，占一般公共预算支出的72.6%。脱贫攻坚有力推进。切实抓好“七个一批”精准扶贫精准脱贫措施，深入开展“挂包帮”“转走访”，筹集各类扶贫资金95.4亿元，实施整乡推进13个、整村推进98个，建设宜居农房1万户、易地搬迁5652户，实施农村危房改造和抗震安居工程2.6万户，减少建档立卡贫困人口53977人，66个贫困村和6个贫困乡出列，贫困发生率从2015年的5.73%降为3.75%，禄劝县达到脱贫摘帽标准。社会保障不断加强。切实做好化解过剩产能企业职工安置、高校毕业生就业等工作，新增城镇就业13.5万人，城镇登记失业率为3.11%。农村低保标准与扶贫标准两线合一，社会保险参保覆盖率达96%。启动建设城乡社区居家养老服务中心38个，新增养老床位3047张。建成保障性安居工程2万套，分配公租房14159套。社会事业明显进步。盘龙、石林等6县区通过国家验收，实现义务教育均衡。组建昆明铁道职业技术学院。引进名校工作取得突破，北京世青、青苗等学校来昆开办5所国际学校，清华附中、黄冈中学、衡水中学等5所学校在昆合作开办11所分校，13所学校与北京建立合作办学机制。资助贫困家庭学生30.4万人次，补助资金达4亿元，适龄少年儿童就学得到有效保障。引进北京中医医院、中日友好医院等8家医院在昆合作办医，新昆华医院建成使用，云南阜外心血管病医院基本建成，优质医疗资源不断扩大。完成7个乡镇卫生院、61个村卫生室标准化建设。全面两孩政策有序实施，人口自然增长率为6.21‰。成功举办上海合作组织昆明国际马拉松赛、高原国际半程马拉松赛、滇池中华龙舟大赛等重大赛事，进一步提升了昆明的国际形象。社会大局和谐稳定。创新立体化社会治安防控体系，推行村（社区）网格化服务管理，县乡村三级矛盾纠纷调解组织作用充分发挥。严厉打击黑拐枪、黄赌毒、网络犯罪、金融诈骗等犯罪活动。组建“春城骑警”，快速反应处置能力得到提升，获得市民好评。安全生产形势总体稳定，较大事故起数下降55.6%。人民群众安全感、幸福感进一步提升。

（七）推进市区融合发展，滇中新区建设提速。市区一体化发展步伐加快，滇中新区一年打基础目标任务圆满完成，实现“1+1>2”的作用。体制机制全面融合。积极构建昆明统筹、新区管理的规划管理体系，协同编制了重点片区等40余项专项规划。理顺市、新区、三县（市）区财政管理体制，国土、环保等7个部门加挂新区管理机构牌子，原来由新区管理的908项社会管理职责全部移交昆明市。产业建设协同推进。东风云汽搬迁技改、昆明新能源汽车工程技术中心等一批重大项目落户新区，浙商产业园、中关村科技产业园启动建设，临空产业园建设稳步推进。基础设施共建共享。实施嵩昆路、机场北高速等69个综合交通基础设施项目，长嵩大道、新320国道（空港段）等20条道路建成通车，新区骨架路网逐步成型成网，综合交通基础设施建设投资完成216.4亿元、增长230%，新区规模以上固定资产投资完成717.1亿元、增长32.7%。

各位代表！六年来，市第十三届人民政府在省委、省政府和市委的坚强领导下，在市人大及其常委会和市政协的监督支持下，全面贯彻党的十八大和十八届三中、四中、五中、六中全会精神，深入学习贯彻习近平总书记系列重要讲话和考察云南重要讲话精神，统筹推进“五位一体”总体布局，协调推进“四个全面”战略布局，努力当好全省经济社会发展的排头兵和火车头，圆满完成了本届政府确定的各项目标任务，为在全省率先全面建成小康社会奠定了坚实基础。

我们聚焦产业发展，聚力结构调整，综合实力不断增强。突出产业强市，加快转型升级，新型工业化提速，现代服务业壮大，高原都市现代农业蓬勃发展。地区生产总值年均增长10.9%，实现翻番。一般公共预算收入、规模以上固定资产投资、社会消费品零售总额实现倍增。三次产业结构由2010年的5.5∶42.4∶52.1调整为4.7∶38.6∶56.7，服务业成为经济转型的新引擎。培育了近50家产值超10亿元的企业，云南白药、云内动力等一批企业成长为行业龙头。官渡区经济总量在全省县区率先突破千亿，安宁市进入全国县域经济百强县，高新区、经开区跻身千亿园区行列，安宁工业园成为全省首个千亿级产能工业园区。

我们致力城乡统筹，促进协同并进，城乡面貌日益改善。优化城市布局，强化基础支撑，加强城市管理，城乡建设取得新进展。呈贡、晋宁撤县设区，滇池国际会展中心、北部山水新区等城市片区加快崛起，城市发展迈进“滇池时代”。以综合交通为重点的基础设施取得重大突破，绕城高速内环实现闭合，“环线+射线”骨干路网基本建成，地铁运营里程达64.3公里，沪昆高铁、云桂铁路建成通车，昆明进入高铁时代，长水国际机场成为国家第四大门户枢纽机场，区域性国际综合枢纽初具雏形。美丽宜居乡村建设深入推进，实现乡乡通油路、村村通公路、乡乡通公交。常住人口城镇化率从64%提高到71.05%。荣获“国家卫生城市”“国家节水型城市”称号。

我们主动服务国家战略，拓展开放格局，战略地位更加凸显。致力深化国际国内区域合作，打造开放平台，提高对外开放的层次和水平，昆明成为“一带一路”重要节点城市、国家对外开放战略前沿。滇中新区成为国家级新区，杨林工业园、阳宗海风景区升级为国家级园区。昆曼国际大通道全线贯通，中缅油气管道全面建成，南博会、旅交会等平台影响力日益扩大。孟加拉国等7国在昆设立领事馆，与印度加尔各答等21个城市缔结国际友城，南亚东南亚友城数位列全国第一。昆明的国际知名度、美誉度、影响力显著提升。

我们坚持问题导向，全面深化改革，发展活力竞相迸发。落实中央顶层设计，围绕发展难点、群众关切，蹄急步稳推进改革，一些重点领域和关键环节改革取得重大成效，形成了全国公共资源交易体制改革的“昆明样本”、基层公共文化服务包的“昆明模式”、户籍制度改革的“昆明做法”、医师多点执业的“昆明实践”、社区治理和服务创新的“昆明探索”。呈贡中小城市综合改革等18项改革任务列为国家级试点。全民参保登记工作经验在全国推广。国企分类监管、薪酬制度、混合所有制改革走在全国前列。政府权责清单向社会公布，行政审批事项进一步减少，审批效率提速63%。

我们狠抓滇池治理，加强污染防治，生态建设成效明显。坚持生态优先，加强环境保护，走好可持续发展道路。全面实施以“六大工程”为主的滇池保护治理工程，以四个层次为主的截污治污系统基本建成，河道综合整治成效明显，牛栏江—滇池补水工程建成通水，实现了“与湖争水”向“还水于湖”的历史性转变，滇池由重度富营养转变为中度富营养，滇池水质企稳向好。滇池湖滨3600公顷“中国最美湿地”扮靓春城。森林覆盖率达50.55%，成为“国家园林城市”“国家森林城市”。主城区空气质量优良率保持在95%以上，“春城绿”“昆明蓝”已成为城市靓丽名片。

我们着力补齐短板，加大惠民力度，群众福祉稳步提升。持续加大民生投入，办好惠民实事，一般公共预算支出的70%以上用于民生。城乡居民收入年均分别增长11.5%和13.7%，高于经济增速，收入比从3.3∶1缩小到2.93∶1。举全市之力打好脱贫攻坚战，23万贫困群众摆脱贫困，贫困发生率从15.2%下降到3.75%。累计新增城镇就业75万人、农村劳动力转移就业135万人次。学前三年教育基本普及，义务教育均衡化加快发展，民办教育突破发展。四级公共文化场所和服务项目全部免费开放。覆盖城乡的公共医疗卫生体系基本建立。统筹城乡居民的基本医疗保险制度、大病保险制度、基本养老保险制度实现全覆盖。解决了105万住房困难人员的住房问题，城镇住房保障率高于全国平均水平。军民融合发展，连续保持“全国双拥模范城”荣誉。社会治安防控体系建设持续加强，安全生产、食品药品监管体系不断健全，社会大局保持和谐稳定。

我们加快转变职能，持续改进作风，服务环境逐步改善。坚持依法行政、从严治政、廉洁勤政，政府自身建设不断加强。依法接受市人大及其常委会的法律监督和工作监督，主动接受市政协的民主监督，积极接受社会监督。累计提请市人大常委会审议制定18件、修订20件、废止3件地方性法规，累计办理人大代表建议1837件、政协提案2867件，制定修订政府规章34件。持之以恒纠正“四风”，深入践行忠诚干净担当。“政府提效率，企业增效益”活动深入开展。加强行政监察、审计监督，严肃查处违纪违法行为，一批腐败分子受到严惩。民族宗教、防震减灾、档案史志、港澳台侨、社会救助等事业取得新进步。

各位代表！奋斗充满艰辛，成绩来之不易，这是党中央国务院亲切关怀的结果，是省委、省政府和市委坚强领导的结果，是全市干部群众团结奋斗的结果。在此，我代表市人民政府，向全市各族人民，向市人大代表和政协委员，向各民主党派、工商联、无党派人士、各人民团体和社会各界人士，向中央驻昆单位和省级各部门，向驻昆解放军和武警部队官兵，向关心支持昆明发展的各界友人，表示衷心的感谢和崇高的敬意！

在充分肯定成绩的同时，我们也清醒地认识到，昆明的经济社会发展还存在许多困难和问题，主要表现在：一是经济总量不大，发展速度不快，创新能力不强，与一些省会城市相比，工业投资占比较小，新兴产业发展滞后，经济总量差距持续拉大。二是城市规划水平不高，重形式轻内涵、重编制轻执行，城市建设缺乏统筹谋划、功能布局不合理、特色不鲜明，基础设施欠账较多，城市管理较为粗放，数字化管理、精细化管理水平不高，交通拥堵等问题突出，马路市场、乱搭乱建等现象依然存在，一些市民的文明素质有待进一步提高，创建全国文明城市工作仍有较大差距。区域发展不平衡，城乡发展差距大，资源环境约束趋紧，滇池保护治理任重道远。三是政务服务环境有待进一步优化，功能和流程不完善，行政审批尚未真正实现并联审批，“互联网+政务服务”推进缓慢，企业、群众办事难的问题仍然突出。四是社会事业发展滞后，公共服务供给总量不足，脱贫攻坚任务艰巨，改善民生面临新的挑战。社会治理水平不高，群防群治工作薄弱，社会矛盾多发，维护社会安全稳定压力较大。

特别是作风建设还有差距，政府自身建设仍需大力加强。有的干部争先进位意识淡薄，创新意识不强。有的干部不作为慢作为不会为，“推、拖、滑、绕、躲”等现象依然突出，不愿担当、不敢担当，抓落实不力。有的干部法治意识不强，运用法治思维和法治方式管理经济社会事务水平不高。有的干部纪律意识、规矩意识不强，“四风”问题禁而不绝，一些领域腐败问题时有发生。对此，我们必须高度重视，直面问题，认真整改，欢迎人大代表、政协委员监督，全力以赴做好政府工作，不负人民重托。

二、 今后五年的基本思路和目标任务

今后五年是昆明在全省率先全面建成小康社会决战决胜的关键时期。习近平总书记考察云南重要讲话，为昆明发展提供了根本遵循和行动指南。李克强总理到云南考察，对昆明工作作出重要指示。省委、省政府要求昆明当好全省经济社会发展排头兵、火车头。市第十一次党代会绘制了建设立足西南、面向全国、辐射南亚东南亚的区域性国际中心城市的宏伟蓝图。我们必须牢记使命，勇于担当，砥砺奋进，努力把宏伟蓝图变成美好现实，谱写昆明跨越发展的新篇章。

今后五年政府工作的指导思想是：高举中国特色社会主义伟大旗帜，以邓小平理论、“三个代表”重要思想和科学发展观为指导，深入贯彻习近平总书记系列重要讲话和考察云南重要讲话精神，坚持“五位一体”总体布局和“四个全面”战略布局，贯彻创新、协调、绿色、开放、共享“五大发展理念”，主动服务和融入国家发展战略，当好云南经济社会发展的排头兵和火车头，在全省率先全面建成小康社会，加快建设立足西南、面向全国、辐射南亚东南亚的区域性国际中心城市。

今后五年经济社会发展主要目标是：到2021年，全市地区生产总值达到7000亿元以上，城镇常住居民人均可支配收入年均增长9%，农村常住居民人均可支配收入年均增长10%。确保2020年以前，全市贫困人口全面脱贫，在全省率先全面建成小康社会。通过五年的努力，全市开放程度明显提高，综合实力明显增强，区域引领、带动和辐射作用明显提升，为建设区域性国际中心城市奠定坚实基础。

为实现上述目标，我们要紧紧围绕市委提出的“打造一个枢纽、当好两个龙头、搭建三个平台、建设四个中心、提升五个品牌”的战略部署，重点抓好七个方面的工作：

一是加快建设区域性国际经济贸易中心和金融服务中心，当好全省发展的火车头。坚持进位争先、跨越发展，推进“188”重点产业发展，在全省的经济首位度提升到三分之一，在全国省会城市排名只进不退。实施“4+4”工业转型升级行动计划，构建工业发展新体系，打造国家先进制造业基地。加快发展大健康、大旅游、大文创产业，打造中国健康之城、国际旅游城市、西部最具竞争力的文化创意之都。做大做强跨境人民币结算业务，提升金融服务能力。积极发展大数据、大服务，推动服务业扩量增质，建设面向南亚东南亚的现代服务业高地。大力发展高原都市现代农业，走出一条市场开放、产出高效、产品安全、资源节约、环境友好的高原都市现代农业发展道路。

二是加快建设区域性科技创新中心，当好全省创新驱动的新引擎。加快科技创新，扩大科技开放，对接国际国内创新资源，推动双创蓬勃发展，促进产业链、创新链、人才链、资金链、政策链集聚，创建高新区国家自主创新示范区，建设国家创新型城市。依托产业集聚人才，创新机制培养人才，优化环境留住人才，打造全省人才集聚新高地。深化供给侧结构性改革，推动重点领域和关键环节改革取得突破，构建有利于加快发展的体制机制，在全省全面深化改革中发挥引领示范作用。

三是加快建设西南开放门户，打造面向南亚东南亚的开放新高地。主动服务和融入“一带一路”、长江经济带国家战略，构建对内对外开放新格局，当好引领云南和我国西南地区与南亚东南亚开放合作的龙头。深化与南亚东南亚地区经济、社会、文化交流，密切与京津冀、长三角、珠三角、成渝等重点区域合作。辐射带动滇中城市经济圈一体化发展，逐步形成“昆明服务+周边制造”模式。深入推进与滇中新区融合发展，共同打造全省重要的经济增长极。加快昆明综合保税区等开放功能区建设，加强国际友城务实交流合作，提升南博会等重大展会功能。积极构建区域性公共事务平台、商务活动平台、协调联动平台，提升昆明在我国与周边国家交往合作中的地位和影响。

四是加快建设区域性国际综合枢纽，打造和谐宜居的现代化城市。坚持量水发展、以水定城，统筹城市核心区、功能拓展区、生态涵养区协同发展，构建“两核一极两区六廊”城市格局。打好“五网”建设五年大会战，高速公路通车总里程超过1000公里，实现市内县县通高速、滇中城市双高速。建成地铁1至6号线和9号线，运营里程超过236公里，力争实现250公里，地铁网络基本形成。建设长水机场综合交通枢纽，推动通用航空产业综合示范区发展，打造国际航空都会。统筹建设油气管网、输配电网络，形成跨区域能源枢纽。推进“云上云”行动计划，完善信息基础设施，建成区域性国际通信枢纽。构建以滇中引水为骨干、中小型水源工程为支撑、水系连通工程为纽带的供水安全保障网。加强城市管理和服务体系智能化建设，提升城市治理水平。

五是加快建设区域性人文交流中心，彰显历史文化名城魅力。实施文化品牌战略，加快文化产业转型升级，大力发展广播影视、数字创意、云媒体服务等新业态，推动文化创意产业与旅游度假、会展博览等产业融合。加大历史文化名城保护力度，加强非物质文化遗产保护传承。健全现代公共文化服务体系，推进文化馆、图书馆、博物馆、体育馆、科技馆建设。提高市民文明素质和城市文明程度，争创全国文明城市。加大城市推介力度，提升文化影响力。

六是加快建设世界春城花都，打造宜居宜业的魅力家园。大力开展城乡园林绿化，深入实施植树造林、退耕还林、天然林保护工程，加强“五采区”、石漠化和水土流失区域生态治理，全面实施大气、土壤、水污染防治行动计划，实现生态环境明显改善。滇池保护治理取得突破，外海水质稳定达到Ⅳ类，草海水质稳定达到V类，湖体富营养水平明显降低。深入开展生态修复城市修补工作和“四治三改一拆一增”“七改三清”环境整治，改善城乡人居环境。争创国家生态园林城市、中国人居环境奖、国家环保模范城市及国家生态文明建设示范区。

七是加快推进城乡统筹发展，持续提升人民群众获得感幸福感。加大对贫困人口和贫困区域的扶持力度，贫困县区如期脱贫摘帽。建立增收脱贫长效机制，确保脱帽不脱政策、脱贫不脱帮扶，巩固脱贫攻坚成效。全面改善农村生产生活条件，自然村公路通达率达到100%，城乡公交覆盖率达到100%。实施更加积极的就业创业政策，年均新增城镇就业10万人以上。织密社会保障网，建立城乡一体的社会保障体系。依法推进公益和慈善事业健康发展。健全住房保障制度，新就业职工住房困难问题得到有效缓解，外来务工人员居住条件得到明显改善。努力办好公平普惠、优质多样、充满活力、人民满意的教育，构建与区域性国际中心城市相匹配的教育体系。坚持健康让生活更幸福的理念，加快建设中国昆明大健康产业示范区，力争综合医疗水平达到西部一流。推动民族地区加快发展，成为民族团结进步示范区建设的引领和标杆。

三、 2017年的主要工作

今年是全面贯彻落实省第十次党代会、市第十一次党代会精神，开启建设区域性国际中心城市新征程的关键一年。我们要按照中央经济工作会、省委十届二次全会、市委十一届二次全会的安排部署，坚持稳中求进、进中求快、快中求好，以推进供给侧结构性改革为主线，以“发展动能转换、主导产业培育、城乡品质提升、民生福祉增进、软硬环境优化、美好家园共建”六大行动为抓手，持续改进作风，狠抓工作落实，奋力推动区域性国际中心城市建设迈出坚实步伐。

今年经济社会发展主要预期目标建议为：地区生产总值增长9%左右，一般公共预算收入增长5%以上，规模以上固定资产投资增长12%，社会消费品零售总额增长12%，城镇和农村常住居民人均可支配收入分别增长8.5%和9.5%，居民消费价格涨幅控制在3%左右，城镇登记失业率控制在4%以内，人口自然增长率控制在7.2‰以内，完成省下达的节能减排目标任务。

围绕上述目标，应重点抓好以下十个方面的工作：

（一）打好工业经济攻坚战，大力振兴实体经济

坚决稳住存量。全力支持云铜、云冶、昆钢等优势企业优化产品结构，确保有色冶金行业增长7%。支持和鼓励磷复肥生产企业产品向专用肥、特种肥方向发展，确保化工行业增长5%。抓好烟叶复烤企业生产调度，力争烟叶复烤行业增长10%，确保烟草行业增速与2016年持平。

积极扩大增量。新增52户新建投产入规企业，培育80户小升规企业，抓好50个以上新建项目投产并形成增量，确保中石油云南炼油项目上半年投产。实施100项重点技改项目、100项重点工业和信息化投资项目，完成工业投资664亿元。加快以食品、饮料、农特产品等为重点的非烟轻工产业转型，确保增长17%以上。推动昆明新能源汽车工程技术中心、东风云汽搬迁技改项目建设，支持3D打印、高端数控机床等智能制造装备发展，装备工业增长15%。

夯实园区载体。强化工业园区基础设施建设，完成基础设施投资120亿元。支持工业园区引入国内外战略投资者、专业化园区运营商合作办园。提高园区发展质量，加大招商履约率落地率、税收对财政贡献率、规模工业增加值占比、新增工业项目投入产出等指标的考核力度。支持园区管理模式改革，激发园区活力。力争园区规模以上工业增加值增长8.5%。

实施精准帮扶。市领导结对帮扶100户重点企业，建立市、县两级联动机制，降低企业停产减产面。扩大市级工业引导基金规模，形成不低于10亿元的资金池，采取“债权+股权”等方式，滚动扶持企业发展。用好小微企业应急贷款周转资金，支持小微企业按时还贷、续贷。完善项目审批绿色通道制度，强化用地保障，降低工业用地成本。新增规模以上工业企业60户，确保规模以上工业增加值增长7%以上。

（二）打好服务经济攻坚战，推动服务业提质增效

着力发展大健康大旅游大文创大数据。抓好大健康产业发展规划组织实施，积极推进部省市共建“中国昆明大健康产业示范区”，加快宝相健康产业园等重点项目建设，确保大健康产业增加值增长15%。深入推进全域旅游，开发高铁旅游产品，加强智慧旅游基础建设。5月中旬开工建设万达文化旅游城和昆明未来城项目，总投资600亿元以上，古滇文化旅游名城项目力争完成投资100亿元，推动环滇池旅游文化健康项目陆续开工。抓好轿子山风景区等68个项目，推进石林国家全域旅游示范县建设，提升世博园、民族村等景区品质，重拳整治旅游市场秩序，确保旅游总收入增长30%。深入实施文化建设和产业发展“510工程”，推进国家文化消费试点城市建设，抓好春雨路沿线老旧厂区转型等项目，确保文化创意产业增加值增长15%。深入推进“云上云”行动计划，加快呈贡信息产业园、中关村电子城（昆明）科技产业园等重点项目建设，确保浪潮云计算产业园投入运行，启动“云上小镇”二期三期建设，确保信息产业规模突破200亿元。

加快服务业载体建设。完善商业网点布局，推进老螺蛳湾片区、东风广场中央商务区等商业集聚区开发，推动电子商务进农村、进社区。分类推进商品交易市场疏解，完成明波家具市场、东聚五金机电建材城等18个市场搬迁，合理布局农贸市场。推动在昆设立人民币对非主要国际储备货币外汇交易中心，组建昆明农商银行。加快呈贡新区、巫家坝片区、北京路沿线等总部楼宇集聚区建设，争取一批国内外合作机构、企业总部、科研机构落户昆明。

创新服务业发展机制。建立“十三五”服务业综合改革试点推进机制，细化落实试点方案，确保按期完成试点任务。加大对服务业的支持力度，整合财政扶持资金，设立服务业发展引导资金，放宽市场准入，落实税收、价格等优惠政策，强化土地、人才等要素保障，为服务业发展营造良好环境，提升“昆明服务”品牌影响力。

（三）突出项目支撑，着力扩大有效投资

强化招商引资。把招商引资作为跨越发展的重中之重、政府工作的第一要事，解决好拿什么招商、招什么样的商、谁来招商、如何招商等问题。落实招商引资奖励政策，注重精准招商、以商招商、中介招商、产业链招商，瞄准世界500强、中国500强，争取引进一批大企业、大项目，确保引进市外到位资金1000亿元以上，实际利用外资8亿美元以上。

突出项目带动。抓好项目前期工作，跟踪对接国家和省产业政策和投资方向，充实完善三年滚动投资计划，形成5000亿元投资规模的项目库，建成PPP项目申报动态储备库。充分利用昆明合作基金、产业发展基金、以购代建基金，继续安排25亿元“五网”建设专项资金，积极争取中央预算内投资项目资金、国家专项建设基金、省重点项目投资基金等资金支持。加强前三批鼓励社会资本投资项目跟踪管理，发布第四批项目，吸引社会资本加大投入。以产业转型、基础设施、公共服务等为重点，实施市级重点项目325个。落实好国家和省稳定房地产市场的各项政策，确保房地产投资完成1650亿元以上、增长8%以上。

推动项目落地。优化项目审批流程，提高项目审批效率。完善投资包保责任制、项目会办制、现场办公制等制度，有效破解项目建设难题，合理布局项目新增建设用地，依法加快土地出让，抓好纳入省“四个一百”、市级重点项目建设。制定项目开工年度计划，以开工时限倒逼前期工作，力争计划新开工项目在三季度前全部开工。

（四）打好脱贫攻坚战，提升城乡协调发展水平

深入实施精准扶贫精准脱贫。按照脱贫攻坚高质量、增收可持续的要求，认真开展“挂包帮”“转走访”，落实好“七个一批”工作举措，整体推进贫困地区脱贫攻坚，完成2.1万户农村危房改造和抗震安居工程建设，启动实施易地搬迁4111户、宜居农房建设3万户以上。计划寻甸县退出贫困县名单、6个贫困乡和67个贫困村出列、6万建档立卡贫困人口脱贫。

加快发展高原都市现代农业。推进农业供给侧结构性改革，稳定粮食生产，保障菜篮子供应，提升畜牧、烤烟、中药材等优势特色产业发展质量，增加绿色优质农产品供给。支持组建“云花集团”，做大做强“云花”产业。新建宜良冬林苑等8座以上都市农庄，建设马金铺现代农业示范园等5个以上农业园区。扶持发展农民专业合作经济组织15个，实现农业龙头企业销售收入640亿元以上。

加速新型城镇化建设。深入推进呈贡区国家中小城市综合改革试点，完成安宁、东川、宜良统筹城乡发展试点工作。新增城镇户籍人口10万人，户籍人口城镇化率达到65%。启动农村公路硬化工程800公里以上、永久性保留自然村公路路基改建工程500公里以上。新建气象公共信息电子显示屏600块，覆盖所有乡镇、行政村和60%以上自然村。完成200个美丽宜居乡村建设任务，推进80个左右美丽宜居乡村省级重点村建设，争取20个村列入国家级传统村落名录、20个村进入省级示范村名录，创建2个民族团结进步示范乡镇、5个民族团结进步示范村（社区），建成18个特色小镇和历史文化名镇。

（五）更加注重改革开放，增强发展活力

推动供给侧结构性改革。积极稳妥去产能，完成国家和省下达的去产能目标任务，巩固钢铁、煤炭去产能成果。更多运用市场化、法治化手段，推动企业兼并重组、破产清算、调整产品结构，有效处置“僵尸企业”。发展租赁市场，激发外来购房需求，有序化解商品房库存，销售面积超过1200万平方米。严格政府债务余额限额管理，加大债务置换力度，优化债务期限结构，降低融资成本。深入开展降低企业成本行动，力争全年电力用户市场化交易电量超过200亿千瓦时，为昆明企业节约用电成本30亿元以上。安排1000万元专项补助资金，对重点企业给予物流运费补助。落实减税降费政策，进一步降低生产要素和制度性交易成本，符合条件的工业项目免缴坝区耕地质量补偿费。聚焦基础设施、公共服务、脱贫攻坚、生态环境等领域，加大投入，补齐短板，满足群众多样化需求。

深化重点领域和关键环节改革。深化国有企业改革，推进市政公用企业健康发展，完善投融资公司资本布局，明确管理服务类企业功能定位，转变产业类企业发展方式，推动国有资本证券化、企业股权多元化，稳步发展混合所有制经济。深化财税体制改革，实施新一轮市对县财政管理体制，探索在县区实行国地税联合办税工作机制。基本完成农村土地承包经营权确权登记颁证工作，抓好农村土地所有权承包权经营权“三权分置”改革。加快国家集体林业综合改革试验示范区建设，完成国有林场改革任务。推进农业水价综合改革。

积极扩大对外开放。确保昆明综合保税区封关运行，高新保税物流中心、腾俊国际陆港保税物流中心通过封关验收。统筹推进铁路口岸规划与申报、跨境电商综合试验区创建、省级服务外包示范城市争创等工作。落实进出口贸易政策，实现进出口贸易总额增长10%以上。新缔结国际友城1座，新增1个国家在昆设立签证中心，争取澜湄旅游城市合作联盟及中国—上合组织青年交流中心落户昆明。配合省办好“2017商洽会”、国际旅交会。

推动创业创新。开展科技创新要素集聚和科技创新产业培育工程，组织开展重点科技创新项目100项。实施科技创新平台倍增计划，新增重点实验室8个、工程技术研究中心15个、院士工作站5个、产业技术创新战略联盟6个，新增各级企业技术中心30家以上。新增高新技术企业80家。深入推进小微企业创业创新基地城市示范建设，创建2个新型双创孵化园，科技企业孵化器新增面积50万平方米。实施科技服务业发展倍增计划，培育300个、引进30个科技服务机构。开展质量提升行动，加强全面质量管理，创建成为全国质量强市示范城市。全面落实促进民营企业发展、民间投资扩大的政策措施，加强民营企业合法权益保护，推动民营企业梯队成长，提振民营企业发展信心。

（六）打好“五网”建设大会战，强化基础支撑

推进综合交通建设。加快福宜、寻沾、武倘寻等16个高速公路项目建设，绕城高速宜良至澄江段建成试通车，昆明至玉溪率先实现双高速。完成鸣泉收费站外迁工程，实现昆玉高速主城至马金铺免费通行。启动建设金马路延长线等102条城市道路，开工建设古滇路官渡段、昆武高速入城段等城市地下综合管廊。加快地铁4、5、9号线等11个在建项目建设，确保3号线、6号线一期投入运行，形成“十字”主干线网。配合做好渝昆高铁开工建设，完成昆明南站配套设施收尾工作。启动建设长水机场综合交通枢纽，全力支持第二航站楼建设。

推进能源和信息基础设施建设。建设19个充电站、2400个充电桩。全面完成主城区天然气置换，启动燃气下乡工作，加快富民、石林天然气支线建设。加强智慧城市基础设施建设，启动智慧交通、城市一卡通、平安城市、智慧治理等领域的建设。加快无线网络建设，实现全市4G网络城乡全覆盖。推进城市老旧小区、商业建筑光纤入户改造提升。

推进水利工程建设。加快柴石滩水库大型灌区工程建设，完成年度投资7亿元。全力推进6件中型水源工程建设，确保海马箐、真金万水库灌区工程完工。实施大平滩等14件小型水源工程。新建核桃箐等6件重点水源工程。启动10万人农村饮水安全巩固提升工程，建设五小水利工程3万件。

（七）打好城乡人居环境建设攻坚战，努力提升宜居品质

强化规划引领。开展新一轮城市总体规划修编准备工作，完成《昆明2050城市发展战略规划》、“1+2+X”综合交通枢纽规划等重点规划编制，全面推进县区“多规合一”工作。编制昆明市城市设计导则，指导全市城市设计工作。加强城市天际线、生态红线保护，突出城市片区功能定位。确保昆明规划馆新馆建成。

加快重点片区建设。实施翠湖周边提升改造，推进草海、巫家坝、官渡文化生态新城、滇池国际会展中心等重点片区开发建设。提速呈贡新区建设，加快完善新区路网，聚集新区人气、商气。制定支持晋宁撤县设区后的发展政策，推动晋宁区尽快融入主城。加强历史文化街区、文物古迹、古镇名村保护修复与合理利用，推动云南陆军讲武堂、胡志明旧居等文物保护利用，延续城市历史文脉。

提升人居环境质量。强力推进生态修复城市修补和城乡“四治三改一拆一增”，分类推进城中村改造，实施54个旧住宅区、16个旧厂区改造。依法拆除违法违规建筑，完成465万平方米治理任务。完成广福路等39条主要城市道路景观提升改造。推进海绵城市建设，力争列入国家试点。新增城市绿地330.3公顷，实施营造林54万亩。开展第二次污染源普查，整改中央环保督察反馈问题，强化建设工地扬尘管理及机动车污染防治，环境空气质量总体保持优良，守住“昆明蓝”“春城绿”。抓好“七改三清”，加快农村环卫设施建设，开工17个乡镇供水设施项目，县级以上主要集中式饮用水水源地水质达标率达95%，乡镇生活垃圾处理设施覆盖率达到98.9%，78.9%的村庄生活垃圾得到有效治理，每个县区建成1个生活垃圾整治示范乡镇，每个乡镇建成1个生活垃圾整治示范村、卫生户厕示范村、公厕建设示范村。让全市人民群众每年都能看到昆明的新变化、昆明的新进步。

加强城市管理。建立覆盖市、区、街道、社区网格一体化社会治理信息网络。加大“治堵”力度，加快发展智慧交通，优化城市道路交通组织，启动机动车出行诱导系统建设，新增机动车泊位2万个。推进“公交都市”创建，加快构建“快线+普线+支线”公交线网，建设公交专用道17.7公里，中心城区公共交通机动化出行分担率达57%以上。投放公共自行车1.5万辆，规范共享自行车管理，努力解决好市民出行“最先和最后一公里”问题。

加强滇池保护治理。启动十三、十四污水处理厂、新宝象河提升整治等7类滇池治理项目，完成投资33.1亿元以上。全面深化河长制，加大不达标河道综合治理力度，加强支次沟渠综合治理，推行河道生态补偿机制，消除滇池流域河道黑臭现象。实施主城污水处理厂水质提升提标，完善环湖截污东岸、南岸配套收集系统及集镇污水处理站配套管网系统，新建2座容量为3.8万立方米的雨污水调蓄池。

（八）提升基本公共服务水平，持续增进民生福祉

做好就业服务。实施好高校毕业生就业创业促进计划、鼓励农民工返乡创业三年行动计划，做好化解过剩产能行业的职工安置工作。新增城镇就业10万人以上，困难高校毕业生就业率保持100%。开展城乡劳动力职业技能培训3万人。完成农村劳动力转移培训15万人次、转移就业13万人次。

完善社会保障体系。深化医保支付方式改革，开展生育保险和职工基本医疗保险合并试点，扩大城镇职工和城乡居民社会保险覆盖面，各项社会保险参保新增10万人，参保覆盖率保持在96%以上。加大城乡低保、特困人员供养、医疗救助和临时救助力度，做到应保尽保。基本建成城镇保障性安居工程2.1万套，解决5.6万人的住房困难问题。启动建设社区居家养老服务中心30个、民办养老机构4个，新增各类养老床位3000张。

推进公共服务均等化。统筹推进城乡义务教育一体化发展，东川、呈贡、宜良、嵩明、禄劝、寻甸6个县区实现义务教育均衡发展目标。推进名校、名师、名校长“三名”工程建设，引进清华附中、北京八十中、黄冈中学等7所名校到昆明办学，筹办好昆明西南联大研究院附属学校。完善农村留守儿童关爱服务体系，实现符合寄宿规定的农村留守儿童学生80%在校寄宿，初中100%在校寄宿。完善昆明公共文化服务包内容，加大公共文化体育基础设施建设力度，推进县级“五馆”及基层综合性文化服务中心建设。实施贫困地区5000户直播卫星户户通工程。推进农村公共体育基础设施建设，实现城乡体育设施全覆盖，办好第十三届世界龙舟锦标赛、市第十一届少数民族传统体育运动会。完成10个乡镇卫生院标准化建设。推进分级诊疗制度建设，推动医疗资源下沉基层，社区卫生服务机构家庭医生服务签约率达到30%。实施基层中医药服务能力提升工程和中医药发展行动计划、“关爱妇女儿童健康行动”，推进流动人口基本卫生计生均等化服务，加快建设国家医养结合试点城市。

维护社会和谐稳定。加大群防群治工作力度，集中开展打击盗抢骗、电信网络犯罪、涉车犯罪等专项行动，深化反恐、禁毒、防艾等工作，严厉打击校园欺凌、暴力等违法犯罪行为。夯实社区基础治理，完善矛盾纠纷多元化调处机制，压实信访责任，加大信访积案排查化解力度，有序解决群众合法诉求。在主城区50%以上的农贸市场规范配置公平秤，维护市场秩序。大力清理整顿市场环境，让市民满意。进一步夯实安全生产基础，强化企业主体责任，坚决防范遏制重特大事故发生。深入践行社会主义核心价值观，全面推进全国文明城市创建工作。争创国家食品安全示范城市。依法管理宗教事务，促进民族团结进步。推动军民融合深度发展，做好双拥共建工作，支持驻昆部队改革建设。

坚持民生为要，继续办好10件惠民实事。

（九）推动市区融合发展，加速打造全省增长极

完善融合发展机制。建立完善与滇中新区的重大项目推进、投资分析联报、信息沟通等工作机制。强化规划引领，加快推进与新区各专项规划的无缝对接。创新社会治理，统筹做好新区的教育卫生、就业社保、综治维稳等工作，促进新区社会和谐。加快构建与新区差别竞争、错位发展、互促互进的发展新格局。

共同推动项目建设。全方位对接新区基础设施和产业发展，合力推进嵩昆路、哨关路、机场北高速、武易高速和轨道交通等重大项目建设，推动基础设施互联互通、共建共享。举全市和新区之力，合力推进先进装备制造、汽车产业园、浙商产业园等重大项目，着力打造市区融合发展的精品工程、示范项目。

（十）全面加强政府自身建设，更好为人民服务

依法行政，接受监督。把法治理念贯穿政府工作全过程，严格按照法定权限和程序用权履责。自觉接受市人大及其常委会法律监督、工作监督和市政协民主监督，认真执行重大事项向市人大常委会报告制度，切实做好人大常委会审议意见、人大代表建议和政协提案办理工作。提高政府规章立法质量，依法做好政府规章和规范性文件备案工作。完善落实法律顾问制度。持续推进行政执法体制机制改革，推动跨部门、跨行业综合执法，解决多头执法、重复检查问题，形成部门联合、随机抽查、按标监管的一次到位机制。落实行政执法责任制，推行行政执法公示制度。

优质服务，提升效能。按照群众和企业到政府办事“最多跑一次”的理念和目标，构建一流发展软环境，不达目的不罢休。从与企业和人民群众生产生活关系最紧密的领域和事项做起，分批制定“最多跑一次”事项，成熟一批公布一批，逐步实现全覆盖。推进市县政务服务中心“一窗受理、集成服务”改革，实行前台综合受理、后台分类审批、统一窗口出件，避免群众和企业在不同部门之间来回奔波，大力降低制度性交易成本。推进“互联网+政务服务+公共资源交易+综合监管”，全面实现便民服务事项“在线咨询、网上办理、证照快递送达”的“零上门”。加快“昆明政务服务”移动客户端建设，推动更多审批事项和便民服务通过互联网办理。继续精减行政事业收费，加快形成覆盖行政许可、行政处罚、行政征收、行政裁决、行政服务等领域的“一次办结”机制。继续削减行政许可事项，推进政府核准投资项目目录“瘦身”。充分发挥昆明投资服务中心作用，全面实行招商引资项目审批手续全程代办。优化政务服务环境第三方评价指标，拓展评价对象范围，运用好评价结果。加快构建“亲”“清”新型政商关系，健全“政商直通车”机制，积极主动为企业提供“保姆式、大棚式”服务，当好高效“服务员”。

从严治政，保持清廉。牢固树立政治意识、大局意识、核心意识、看齐意识，在思想上政治上行动上始终同以习近平同志为核心的党中央保持高度一致，把党中央国务院、省委省政府和市委各项决策部署落到实处。强化责任担当，营造良好干事创业氛围，建立容错纠错机制，激励想干事的人，保护敢做事的人。严格落实党风廉政建设主体责任和“一岗双责”，强化行政监察和审计监督，坚决惩治公共资源交易、扶贫攻坚、矿产资源、土地出让等重要领域违法违规行为。严格财经纪律，严控“三公”经费，把每一分钱都花在刀刃上，用政府的“紧日子”换取群众和企业的“好日子”。

各位代表！我们骄傲地拥有“昆明蓝”，要同心同德、齐心协力，创造昆明跨越发展的春天。让我们更加紧密地团结在以习近平同志为核心的党中央周围，在省委、省政府和市委的坚强领导下，撸起袖子加油干，凝心聚力抓落实，为在全省率先全面建成小康社会，为把昆明加快建设成为区域性国际中心城市而努力奋斗，以优异成绩迎接党的十九大胜利召开！
